# Totally three-dimensional endoscopic mitral valve replacement in a patient with situs inversus totalis

**DOI:** 10.1093/jscr/rjae583

**Published:** 2024-09-16

**Authors:** Taro Kanamori, Shota Yamanaka, Yohei Onga, Koki Maekawa, Shu Takahashi

**Affiliations:** Department of Cardiovascular Surgery, Kawaguchi Cardiovascular and Respiratory Hospital, 1-1-51, Maekawa, Kawaguchi, Saitama 333-0842, Japan; Department of Cardiovascular Surgery, Kawaguchi Cardiovascular and Respiratory Hospital, 1-1-51, Maekawa, Kawaguchi, Saitama 333-0842, Japan; Department of Cardiovascular Surgery, Kawaguchi Cardiovascular and Respiratory Hospital, 1-1-51, Maekawa, Kawaguchi, Saitama 333-0842, Japan; Department of Cardiovascular Surgery, Kawaguchi Cardiovascular and Respiratory Hospital, 1-1-51, Maekawa, Kawaguchi, Saitama 333-0842, Japan

**Keywords:** situs inversus totalis, mitral valve replacement, totally 3D endoscopic surgery, a 3-port system, minimally invasive cardiac surgery

## Abstract

Situs inversus totalis (SIT) with dextrocardia is a rare congenital anomaly that poses a surgical challenge. This case report presents the first known case of a totally 3D endoscopic mitral valve replacement (MVR), which was performed in a 75-year-old woman with SIT and severe functional mitral regurgitation. Despite the anatomical complexity, the procedure was successfully completed using a simplified three-port system and a 3D endoscope by requiring careful preoperative planning and intraoperative adaptation to the mirrored anatomy of SIT. This case report demonstrates the feasibility and potential benefits of totally endoscopic MVR in patients withSIT.

## Introduction

Situs inversus totalis (SIT) with dextrocardia is a rare anomaly in which all organs are completely mirrored, occurring in ~1 in 10 000 individuals [1]. The prevalence of cardiovascular disease in SIT is similar to that in the general population, but surgical treatment can be challenging due to limited access [2]. Reports of cardiac surgery in patients with SIT are rare, and even fewer reports describe minimally invasive approaches [3–6]. Minimally invasive cardiac surgery has gained popularity and proven its usefulness in recent years [7–9]. Here, we report the first case to our knowledge of a totally endoscopic mitral valve replacement (MVR) using a 3D endoscope without robotic assistance in a patient with SIT.

## Case report

**Figure 1:**
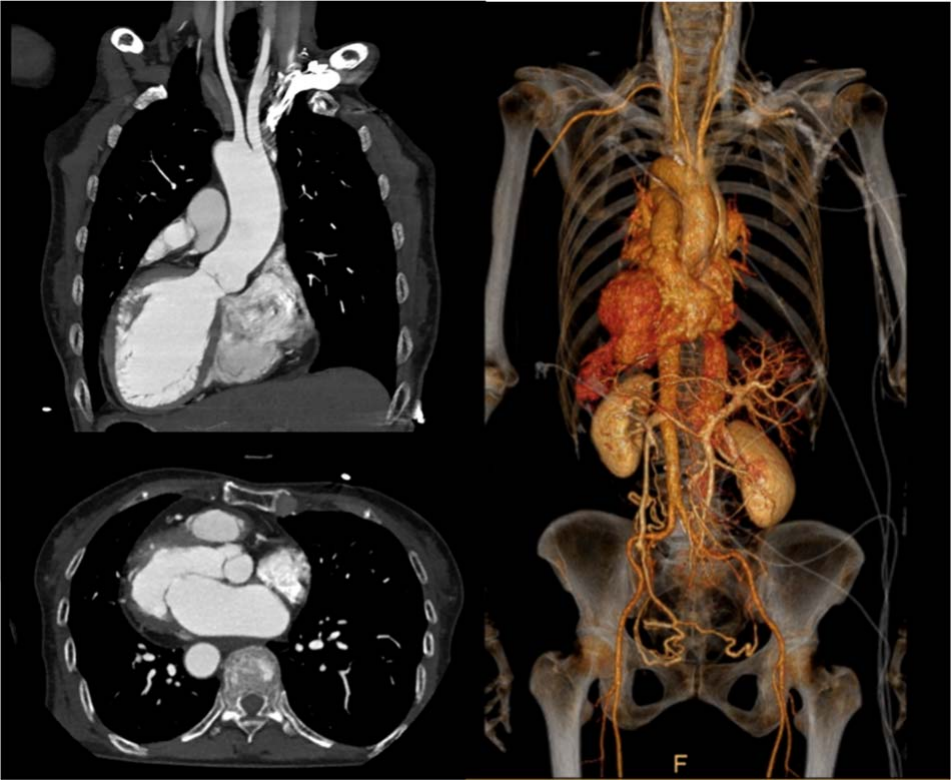
Preoperative contrast-enhanced CT: axial, coronal oblique MIP, and coronal oblique 3D VR images demonstrating situs inversus totalis and dextrocardia.

A 75-year-old woman with a history of SIT and dextrocardia was referred to our department for surgical intervention due to severe functional mitral regurgitation (MR) causing heart failure despite medical therapy. Chest X-ray showed dextrocardia and cardiomegaly with a cardiothoracic ratio of 56.1%. ECG showed sinus rhythm at 75 bpm. Contrast-enhanced computed tomography (CT) showed complete situs inversus with mirroring of all organs and no other congenital anomalies (Fig. 1). Transesophageal echocardiography showed a reduced left ventricular ejection fraction of 43% and severe functional MR due to tethering of both leaflets was also observed.

**Figure 2:**
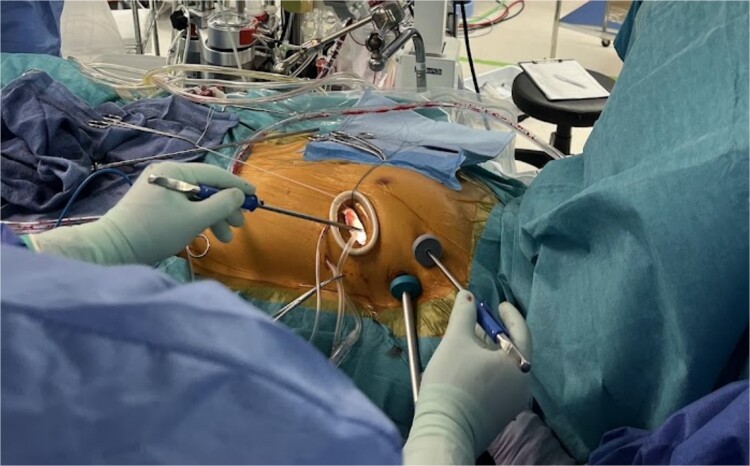
Appearance of the intraoperative surgical field: Setup of the simplified three-port system with 3D endoscopy.

We planned totally endoscopic MVR using a 3D endoscope without robotic assistance. Preoperatively, all equipment was arranged in a mirrored fashion. After double-lumen endotracheal intubation of general anesthesia, a central venous catheter was inserted into the right jugular vein, and a superior vena cava venous drainage cannula and a temporary ventricular pacing catheter were inserted into the left jugular vein. After heparinization, an arterial cannula and an inferior vena cava venous drainage cannula were inserted into the left femoral artery and vein, respectively, to establish cardiopulmonary bypass (CPB). A 4-cm left thoracotomy was performed in the fourth intercostal space (ICS). A 10-mm trocar for the 3D endoscope (Karl Storz) was inserted through the third ICS, and a 5-mm port for right-handed instruments was placed in the second ICS. Left-handed instruments were inserted through the main incision, and the procedure was performed using a three-port system centered on the 3D endoscope (Fig. 2). After cardiac arrest was achieved, the mitral valve was exposed and replaced using standard techniques. The Epic 29 mm bioprosthetic valve was sutured using the Cor-Knot surgical tying device. Weaning from CPB was uneventful (CPB time and cross clamp time was 132 and 84 min, respectively). The patient was recovered uneventfully.

## Discussion

Minimally invasive cardiac surgery has become the first-line surgical treatment for mitral valve disease [7, 10]. In addition, the utility of endoscopic approaches, with or without robotic assistance, has been reported worldwide [11–13]. Excellent valve exposure and precise repair techniques are essential for successful mitral valve surgery. The endoscopic approach allows the surgeon to view the mitral valve from the front and closely observe the mitral valve on a 3D endoscopic monitor image, providing an accurate anatomical understanding of the repair site.

Robotic surgery is currently the mainstream approach to endoscopic cardiac surgery [11]. However, there are also reports on the usefulness of 3D endoscopic surgery without a robotic system [12, 13]. Ito *et al*. [12] reported that totally three-port endoscopic minimally invasive mitral valve surgery was reproducible and safe in 250 consecutive patients. In our case, we also performed a totally 3D endoscopic mitral valve replacement using the simplified three-port system. Compared to robotic-assisted surgery, the three-port system with 3D endoscopy has several advantages. One is its overwhelming cost effectiveness. It is less expensive to implement and requires low running costs and superior from a labor cost standpoint, as a minimum of one doctor was required to perform a surgery, with the assistance of co-medical staffs. The second is the simplicity of preparation: if a 3D endoscopic system is available, no other major equipment is required, and the system can be easily applied to a wide variety of cases. In this case of the built-in inversion, it was easy for us, who are accustomed to 3D endoscopic surgery, to perform symmetrical preoperative preparation.

**Figure 3:**
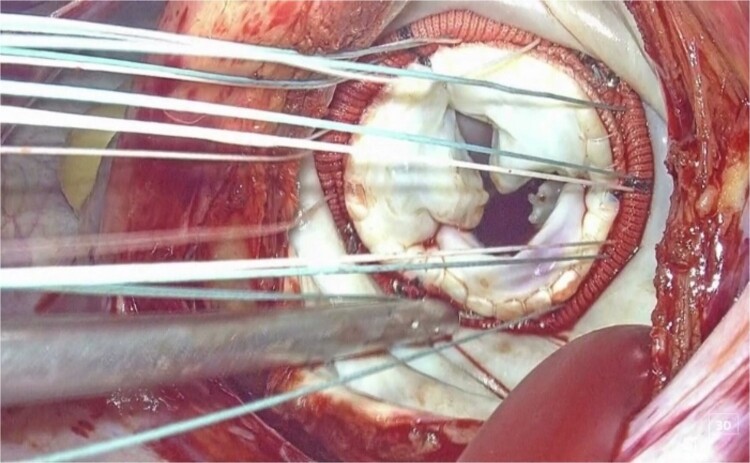
3D endoscopic view of the intraoperative field: Sutures, prosthetic valves, and the Cor-Knot surgical tying device are inserted through the main incision on the left side toward the mitral valve.

**Figure 4:**
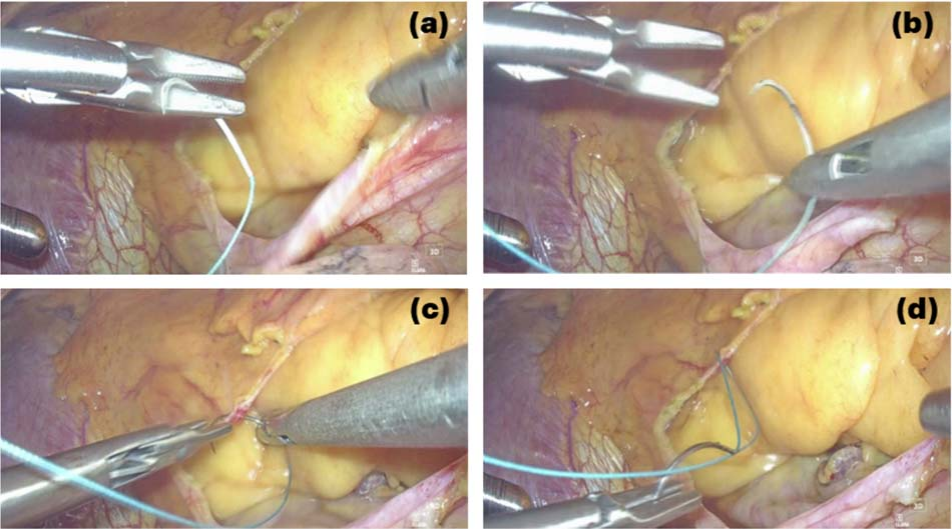
3D endoscopic view of the intraoperative field: We are right-handed, needle thread inserted by using the left-handed forceps is switched to the right-handed needle holder to perform the suture (a → b → c → d).

This is believed to be the first report of a totally 3D endoscopic mitral valve replacement in SIT. We have demonstrated that totally endoscopic surgery is feasible in cases of SIT and that its advantages can be offered to the patient. Several precautions were necessary for the success of this procedure given the anatomy of SIT. The first was the placement of the surgical instruments under the preoperative mirror image. All settings, positions, and instruments must be placed in opposite directions, and the cannulation sites must be completely opposite. The second is to adapt to the inverted left–right anatomy of the thoracic cavity. It is important to note that the main incision in the fourth ICS is on the left side of the mitral valve, contrary to the usual approach. This means that needles and sutures, prosthetic valves, and other devices are inserted through the main incision on the left side toward the mitral valve using a left-handed forceps; if we are right-handed, we must switch the needle threads to a right-handed needle holder in the thoracic cavity for each suture (Figs 3 and 4).

By requiring careful preoperative planning and intraoperative technical adaptation, we were able to successfully perform totally 3D endoscopic MVR in SIT, offering its advantages to the patient.
